# Improvement of the Process Stability of Arylmalonate Decarboxylase by Immobilization for Biocatalytic Profen Synthesis

**DOI:** 10.3389/fmicb.2017.00448

**Published:** 2017-03-16

**Authors:** Miriam Aßmann, Carolin Mügge, Sarah Katharina Gaßmeyer, Junichi Enoki, Lutz Hilterhaus, Robert Kourist, Andreas Liese, Selin Kara

**Affiliations:** ^1^Institute of Technical Biocatalysis, Hamburg University of Technology,Hamburg, Germany; ^2^Junior Research Group for Microbial Biotechnology, Ruhr-University Bochum,Bochum, Germany

**Keywords:** profen, arylmalonate decarboxylase, process stability, immobilization, biocatalysis, enantioselectivity

## Abstract

The enzyme arylmalonate decarboxylase (AMDase) enables the selective synthesis of enantiopure (*S*)-arylpropinates in a simple single-step decarboxylation of dicarboxylic acid precursors. However, the poor enzyme stability with a half-life time of about 1.2 h under process conditions is a serious limitation of the productivity, which results in a need for high catalyst loads. By immobilization on an amino C2 acrylate carrier the operational stability of the (*S*)-selective AMDase variant G74C/M159L/C188G/V43I/A125P/V156L was increased to a half-life of about 8.6 days, which represents a 158-fold improvement. Further optimization was achieved by simple immobilization of the cell lysate to eliminate the cost- and time intensive enzyme purification step.

## Introduction

The integration of biocatalysis is nowadays well-established in chemical industry, especially in the synthetic segments for stereo-, regio-, and enantioselective production of organic fine chemicals and drugs (Schmid et al., 2002). Biocatalysis combines several desired characteristics for an efficient conversion, such as high selectivity and environmentally friendly reaction conditions. However, the implementation of enzymes bears some challenges, like the stability under process conditions and separation of the soluble biocatalyst from the product after the process (Klibanov, 1983). Reaction conditions in industrial applications of biocatalysts often differ from the natural enzyme environment, which can lead to a fast enzyme inactivation. This drawback can be circumvented either by molecular engineering methods, i.e., optimizing the enzyme structure to receive a more stable biocatalyst (Bornscheuer et al., 2012), or by immobilization of the enzyme (Liese and Hilterhaus, 2013).

Several immobilization methods are available and well-established: (i) Immobilization onto a solid carrier material, (ii) enzyme affixed into a matrix with enzyme encapsulation/entrapment strategies or (iii) enzyme immobilized via crosslinking (Hanefeld et al., 2009). In this study, we focus on the immobilization of enzymes on solid carrier materials. The coupling of the enzyme onto the carrier can be realized in a reversible or an irreversible manner. In the irreversible way, the enzyme is coupled to the carrier by a specific covalent binding of amino acid side chains (lysine, cysteine, aspartic or glutamic acids) via stable amide, thioether, or carbamate bonds to the carrier material (Mohamad et al., 2015). For the reversible coupling, different methods are available. The most common ones are achieved through unspecific adsorption by ionic or hydrophobic interaction with the enzyme (Guisan, 2006), disulfide bond formation with cysteine side chains (Simons et al., 2013) or through complexation, e.g., with His_6_ affinity tagged proteins (Engelmark Cassimjee et al., 2014). Beside the bonding type, different carrier properties influence the enzyme immobilization. Especially the carrier size, its porosity and surface properties have a significant impact on the enzyme loading and activity yield (Datta et al., 2013).

**FIGURE 1:**
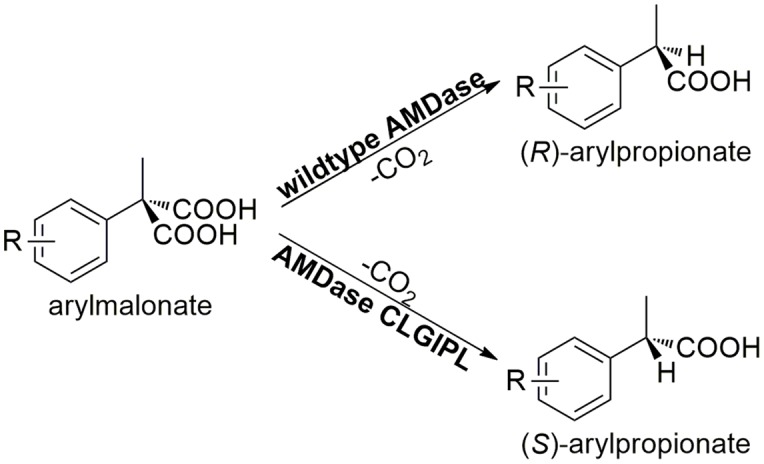
**AMDase catalyzed asymmetric synthesis of (*R*)-arylpropionates (wild type AMDase) and (*S*)-arylpropionates (AMDase-CLGIPL).** R can be arylic, aliphatic, halogen atoms and other substituents.

In this study, we demonstrate the advantage of enzyme immobilization for an efficient synthesis of α-arylpropionate derivatives. These so-called profenes, like ibuprofen or naproxen, are widely used as non-steroidal anti-inflammatory drugs (NSAID) (Rao and Knaus, 2008). The challenge in the synthesis of this class of molecules is their chirality, since only the (*S*)-enantiomers show the desired medical effects (Nguyen et al., 2006). One enzyme capable of catalyzing the crucial enantioselective synthetic step in a selective and environmentally friendly manner is arylmalonate decarboxylase (AMDase, EC 4.1.1.76). AMDase originates from *Bordetella bronchiseptica* and was discovered by Miyamoto and Ohta (1990). Since then, this enzyme received significant attention owing to its capability of a highly enantioselective arylpropionate synthesis. The natural (*R*)-selective wild type enzyme could successfully be designed into a (*S*)-selective mutant through the exchange of crucial amino acids in the active site (Ijima et al., 2005; Kourist et al., 2011). The (*S*)-selective AMDase variant G74C/M159L/C188G/V43I/A125P/V156L (AMDase-CLGIPL) shows an excellent (>99%) enantioselectivity in the production of (*S*)-flurbiprofen (Gaßmeyer et al., 2016). **Figure 1** illustrates the general decarboxylation reaction of arylmalonates catalyzed by the wild type or the AMDase mutant to the respective (*R*) and (*S*)-arylpropionates, respectively. Unfortunately, AMDase is known to be an unstable enzyme with a theoretical instability index of about 43.20 (ExPASy ProtParam tool, Guruprsad et al., 1990). Enzyme immobilization is the method of choice to tackle the stability issues in order to develop a process for the efficient biocatalytic synthesis of enantiopure arylpropionates and hence is the focus of this study.

## Materials and Methods

### Protein Expression and Purification

Recombinant *Escherichia coli* BL21 (DE3) containing an IPTG (isopropyl-β-D-thiogalactopyranosid) inducible plasmid with the gene for AMDase G74C/M159L/C188G/V43I/A125P/V156L (AMDase-CLGIPL) (Gaßmeyer et al., 2016), was cultivated as described in Gaßmeyer et al. (2016). After protein expression, the cells were harvested by centrifugation (20 min, 4°C, and 17,700 ×*g*) and stored at −80°C unless directly used. For cell lysis, the cell pellet was resuspended to a concentration of 0.2 g_cells_/mL in 50 mM Tris buffer pH 7.0 or in water and ultrasonication was performed using a Sonoplus HD2070 sonicator (90% intensity, 3 × 3 min, and interjacent cooling on ice). The cell lysate was separated by centrifugation from the solid cell components (30 min, 4°C, and 74,000 ×*g*). The cell lysate was either directly used or the histidine tagged AMDase was purified over a Ni-NTA HisTrap IMAC (immobilized metal ion affinity chromatography) column. For enzyme purification, 10 mM imidazole was added to the lysate. The resin was equilibrated (50 mM Tris buffer, 10 mM imidazole, and pH 7.0), afterward the lysate was loaded onto the purification column and washed with five column volumes of washing buffer (50 mM Tris buffer, 20 mM imidazole, and pH 7.0). The target enzyme was eluted by increasing the imidazole concentration with elution buffer (50 mM Tris buffer, 250 mM imidazole, and pH 7.0). The collected enzyme fractions were desalted and concentrated using an Amicon ultrafiltration cell equipped with a 10 kDa membrane. The concentration of the obtained protein solution was determined by Bradford Assay using the reported method of Zor and Selinger (1996). Activity based protein analysis was also done to determine the target protein concentration in the cell lysate (Supplementary Figure S6).

### Enzyme Immobilization

Carriers EziG^TM^ 1, 2, and 3 were kindly provided from Enginzyme (Stockholm, Sweden). Amino C2 acrylate carrier was obtained from Iris Biotech GmbH (Marktredwitz, Germany), Trisoperl carrier was purchased from Sigma Aldrich (Steinheim, Germany), and Sepabeads carriers were obtained from Residion S.r.l. (Binasco, Italy). Immobilization of wtAMDase or (*S*)-selective AMDase-CLGIPL on different carrier materials for screening of suitable solid supports was performed with freshly purified enzyme or the cell lysate from freshly lysed cells, respectively. In the case of the amine functionalized carrier, a pre-activation with a 2% v/v glutaraldehyde solution was carried out (212 mM, ratio 1:4 w_carrier_/v, 250 mg/mL), 1 h, overhead shaker, room temperature, 8 rpm), filtered with a sintered glass filter (pore size 2, 40–100 μm) and then carefully washed with distilled water. For immobilization, the different carrier materials were incubated with the enzyme solution (40 mg_carrier_/mL, 4–8 mg_enzyme_/mL) in 50 mM Tris buffer at pH 7.0 (for the first screening experiment with wtAMDase) or in water with pH 8.0 (starting with AMDase-CLGIPL), 17–19 h, overhead shaker at 8 rpm and room temperature. Afterward, the carrier was filtered or separated by centrifugation (4220 ×*g*), washed twice with one volume of Tris buffer (50 mM, pH 7.0) and with one volume of 0.5 M NaCl solution. Fractions of the supernatant and the washing steps were collected for further determination of protein concentration.

### Batch Experiments with the Immobilized Enzyme

For activity analysis, the immobilized enzyme was tested in batch experiments in 50–100 mL stirred tank reactors for the conversion of 20 or 25 mM phenylmalonic acid in 45 mM Tris buffer or in water at pH 7.0 or 8.0 (adjusted with 1 M NaOH), at 200 rpm and 30°C. The reaction progress was monitored by HPLC. Between the batches, the carrier material was carefully washed with distilled water, filtered or separated by centrifugation (5 min, 4220 ×*g*) and stored at 4°C until the next usage.

### Recycling Study in Naproxen Malonate Conversion

The recycling capability of the enzyme was tested for the conversion of naproxen malonate (110 mM) at pH 8.0. 100 mg of the amino C2 acrylate carrier with immobilized purified (*S*)-selective AMDase-CLGIPL were used in a 2 mL round bottom microreaction tube (total reaction volume of 1.5 mL). The reaction was started by the addition of the substrate solution and conversion was monitored by HPLC. Between the batches, the carrier was washed with 1 mL of distilled water, separated by centrifugation (2 min, 12,100 ×*g*) and stored at 4 °C until the next usage.

### Investigation of Process Stability of the Immobilized Enzyme

For stability analysis, 100 mg of amino C2 acrylate carrier containing the target enzyme was incubated in a round bottom microreaction tube (2 mL) with a total reaction volume of 1 mL at 30°C without substrate and shaken at 900 rpm in water at pH 8.0 (adjusted with 1 M NaOH) for desired time intervals (0–600 h). For activity measurements, the immobilized enzyme was separated by centrifugation (2 min, room temperature, 12,100 ×*g*) and the supernatant was discarded. Afterward, fresh substrate (1 mL, 25 mM naproxen malonate, pH 8.0) was added to the immobilized enzyme and the conversion was monitored by HPLC analysis. After the reaction (72 min for the first activity analysis, up to 254 min in the last analysis), the carrier was again separated from the reaction mixture by centrifugation (2 min, room temperature, 12,100 ×*g*), and the supernatant was discarded. The carrier was washed with 1 mL of water at pH 8.0 and incubated under the same conditions (1 mL, 900 rpm, pH 8.0, at 30°C).

### Sampling and HPLC Analysis

Samples (500 μL) for the HPLC analysis of the phenylmalonate conversion were mixed with acetonitrile (250 μL) and transferred directly into the HPLC vials. For the analysis of naproxen malonate conversion, samples (12 μL) were collected and mixed with acetonitrile (6 μL) and diluted 10-fold with water/acetonitrile (1:1). HPLC analysis was realized with a C18 reversed phase column (Nucleodur C18 pyramid 250/4.6, Macherey Nagel) in an Agilent 1100 HPLC system using an isocratic eluent of ACN:H_2_O:TFA (59.025:39.025:0.05). The flow rate of 0.8 mL/min was maintained for 6 min for phenylmalonate conversion or 9 min for naproxen malonate conversion. The detection was carried out with a diode array detector at a wavelength of 245 nm. Typical retention times were: naproxen 5.8 min, naproxen malonate 3.8 min, phenylacetate 4.5 min, phenylmalonate 3.8 min. Reference chromatograms are available in the Supplementary Figures S1, S2.

## Results and Discussion

### Investigation of the Process stability of the Free Enzyme

**FIGURE 2:**
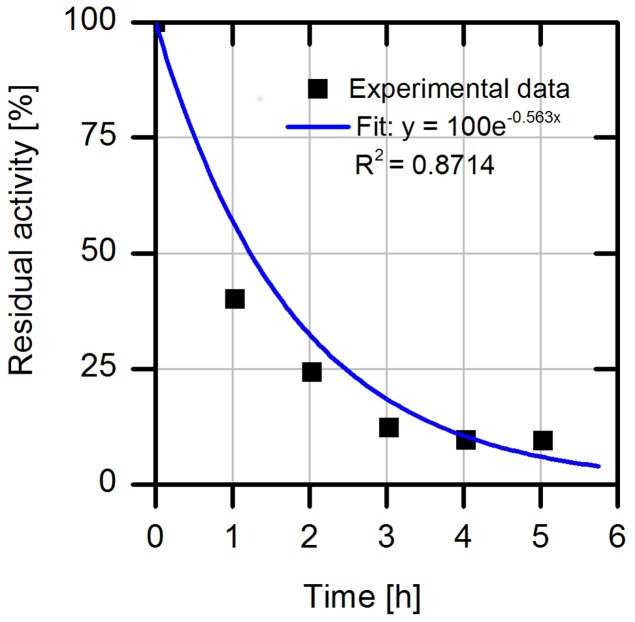
**Relative residual activity of purified free wtAMDase under process conditions with 2 mg/L wtAMDase, 37.5 mM Tris buffer at pH 7.0, 200 rpm and 30°C.** The specific activities were measured by taking samples from the incubation mixture and measuring the initial reaction rates with 20 mM phenylmalonate as substrate.

The operational stability is a central aspect for the application of enzymes in industrial processes. Hence, we first analyzed the process stability of free wild type AMDase (**Figure 2**). The result of the experiment with the purified free wtAMDase reveals a low process stability with a half-life time (t_1/2_) of 1.2 h. The reduction of AMDase activity under thermal incubation over 40°C has already been analyzed by Miyamoto and Ohta (1992) who described a 10% loss of activity at 40°C after 10 min and a 50% activity loss at 50°C with 10 min incubation. These data indicate an enhanced enzyme inactivation in a temperature range over 40°C.

The production of the biocatalyst is an important parameter for the final cost and environmental footprint of the arylpropionate synthesis. Therefore, the enzyme stability needs to be significantly increased in order to reduce the amount of enzyme needed per unit of product. Immobilization is one of the most common methods for enzyme stabilization (Bornscheuer, 2003). The possibility of AMDase immobilization had earlier been demonstrated by using polystyrene nanoparticles, allowing a recycling of the enzyme up to four times (Wong et al., 2010). Since we aimed at significantly improving the stability of the enzyme for profen synthesis, we chose immobilization on a solid support with larger particle size (∼100–400 μm diameter) than nanoparticles for an easy separation and straightforward downstream processing, as described in the next sections.

### Screening of Different Carriers for Immobilization

**FIGURE 3:**
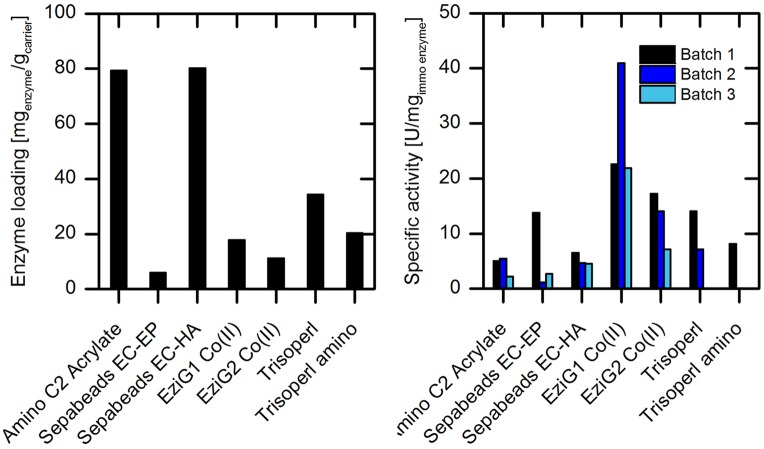
**Investigation of different carrier materials with respect to enzyme loading (left) and specific enzyme activity in repeated batch experiments (right) with immobilized purified wtAMDase.** Reaction conditions **(right)**: 100 mL reaction volume in stirred tank reactor with overhead stirrer, 200–500 mg carrier material, 20 mM phenylmalonate, 45 mM Tris buffer at pH 7, 200 rpm, and 30°C. Between the batch experiments, the carrier was filtered and stored at 4°C.

Screening of solid supports for enzyme immobilization was carried out with different types of carrier materials with regard to (i) surface properties, (ii) bonding type, (iii) spacer length, and (iv) particle size, as these parameters influence the catalytic performance of the immobilized enzyme (Guisan, 2006). In total, seven different carriers were evaluated in this first set of experiments: Amino C2 acrylate, Sepabeads^TM^ EC-EP and EC-HA, EziG^TM^ 1 and 2 with Co(II) loading, Trisoperl^TM^ and Trisoperl^TM^ amino. Detailed information on the specification of the carrier materials are given in Supplementary Table S1 and demonstrate the different characteristic properties each carrier type has. According to the crystal structure of wild type AMDase (PDB entry 3IP8, Supplementary Figure S3) (Okrasa et al., 2009) two lysine resides on the surface of the enzyme are available for crosslinking with the glutaraldehyde activated carrier. Alternately, for immobilization via complexation, there is only one possible immobilization position, the N-terminal His_6_-tag (Supplementary Figure S3), which still provides six amino acids for an efficient immobilization. A comparison of the effectivity of enzyme immobilization on these distinctly different carriers and binding modes is therefore highly desirable. For screening purposes, we first focused our attention on purified wtAMDase. Phenylmalonate was used as a readily available model substrate for determining the specific activity of immobilized enzyme on the different carriers. Measurements were performed by analyzing the initial reaction rates at up to 5% substrate conversion. **Figure 3** shows a comparison of the carrier materials with respect to enzyme loading and activity of the immobilized enzyme.

Among the carriers evaluated, the amino C2 acrylate and Sepabeads EC-HA showed the highest enzyme loadings of about 80 mg_enzyme_/g_carrier_ (**Figure 3**). This is probably due to the high density of functional groups on these carriers with 1760 μmol/g_carrier_ for amino C2 acrylate and >700 μmol/g_carrier_ for EC-HA (Supplementary Table S1). The immobilized enzyme showed activity in the conversion of phenylmalonate on all tested carrier materials with activities of 2% (amino C2 acrylate, 5.1 U/mg_enzyme_) to 8% (EziG1 Co(II), 22.6 U/mg_enzyme_) compared to the purified free wtAMDase with 282 U/mg_enzyme_ (Supplementary Figure S4 and Table S2). In respect to the binding type, the covalently linked enzymes (on carriers of acrylate, EC-EP and EC-HA) reveal the lowest activities. Nevertheless, in the case of the acrylate and EC-HA resin only a slight loss of activity during enzyme recycling was observed. The spacer length, which could in principle have a significant influence on the flexibility and activity of the immobilize enzyme (Cao, 2005), shows no influence on the enzymatic activity (acrylate: C2; EC-HA: C6). However, stability was slightly enhanced with longer spacer length (**Figure 3**). In the repeated batch experiments we observed for the amino C2 acrylate and EziG1-Co(II) carrier enhanced specific activities during the second batch. One of the reasons might be wetting effects. If carriers are not in contact with the reaction solution beforehand, after the first batch higher activities can be observed due to the change of surface polarity. Furthermore, partial substrate adsorption onto the carriers and consequently an increase of the effective substrate concentration for the second batch could occur. Using the amino C2 acrylate and EziG1-Co(II) carriers, 8 and 4% adsorption of phenylmalonate onto the carrier material was observed, therefore making this a possible explanation (Supplementary Figure S7).

The highest catalytic performance was found for the enzyme bound to the porous glass carrier EziG^TM^ Co(II) types 1 and 2 (**Figure 3**). Here the enzyme is linked by coordination of the His_6_ tag to cobalt(II) metal ions. Making use of this affinity tag allows to combine enzyme purification and immobilization in one step (Engelmark Cassimjee et al., 2014).

Whilst the preliminary immobilization assays were conducted with wtAMDase, we chose the purified (*S*)-selective AMDase mutant (AMDase-CLGIPL) for further analysis, as this variant allows the synthesis of the desired (*S*)-arylpropionates with an excellent enantioselectivity (Gaßmeyer et al., 2016). In a first set of recyclability experiments, AMDase-CLGIPL activity was evaluated on different types of porous glass carrier EziG^TM^. The carrier materials differ in surface hydrophobicity (type 1: hydrophilic; 2: hydrophobic; 3: semi-hydrophilic) and in the coordinating metal ion (Co(II) or Fe(III)).

**FIGURE 4:**
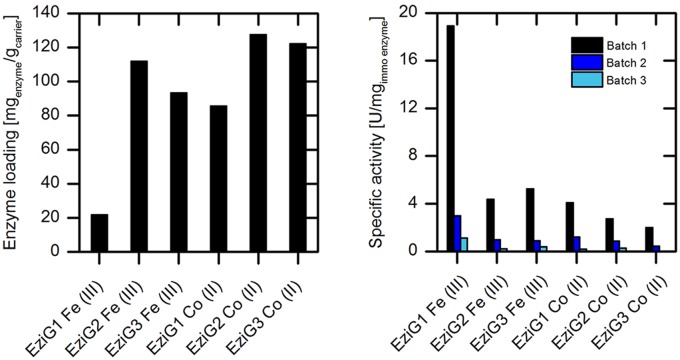
**Investigation of different types of EziG^TM^ porous glass carrier with respect to enzyme loading (left) and specific enzyme activity in repeated batch experiments (right) for phenylmalonate conversion catalyzed by immobilized purified (*S*)-selective AMDase-CLGIPL.** Reaction conditions **(right)**: 50 mL reaction volume, 2 g/L carrier, 45 mM Tris buffer at pH 8.0, 20 mM phenylmalonate, 400 rpm, 30°C. Between the batch experiments, the carrier was filtered and stored at 4°C.

Analyses were carried out with respect to the enzyme loading and specific activity (**Figure 4**). The enzyme loading varied between 80 and 120 mg_enzyme_/g_carrier_, with the exception of EziG^TM^ carrier type 1 with Fe(III), which revealed a low loading of 20 mg_enzyme_/g_carrier_. The enzymatic activity was up to fivefold higher when immobilized on Fe(III)-based carrier was used compared to the Co(II)-based EziG^TM^ carrier material. In respect to the surface properties of the carrier, we observed the best activity results for enzyme bound to the hydrophilic carrier EziG1 for both coordinating metals. Unfortunately, we also observed a significant loss of enzymatic activity with all EziG^TM^ carriers during re-use under the applied reaction conditions. This could be explained by enzyme leakage through the comparatively weaker coordinative binding. A second influencing factor for the observed activity loss might possibly be local pH shifts inside the porous carrier due to the formation of arylpropionates from malonic acids. However, overall pH shifts after the batch experiments were not observed.

### Analysis of Enzyme Recycling in Repetitive Batch Experiments

With the aim to integrate the immobilized enzyme into an arylpropionate production process, further investigations were carried out with (*S*)-selective AMDase-CLGIPL to evaluate the possibility of enzyme recycling and to demonstrate the advantages of enzyme immobilization. For this purpose and based on the above-described results, we now used the amino C2 acrylate carrier for further experiments as “best candidate”. In addition, owing to its large particle size (400 μm), its separation from the reaction solution is straightforward and can be conducted, e.g., by filtration or centrifugation. Carriers such as the significantly smaller Trisoperl material are much more difficult to remove. The relatively large pore size (120 nm) of the amino C2 acrylate carrier additionally facilitates the access of substrate molecules to the immobilized enzyme inside the carrier material and may also reduce the chance of local pH “hotspots” that might result in enzyme inactivation.

**FIGURE 5:**
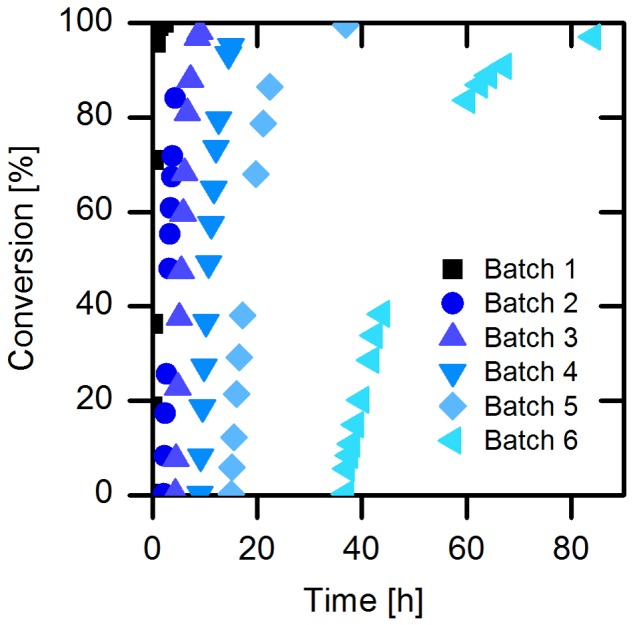
**Conversion of naproxen malonate catalyzed by immobilized enzyme.** Reaction conditions: 1.5 mL reaction volume, 110 mM naproxen malonate, 66.7 g/L amino C2 acrylate with purified immobilized (*S*)-selective AMDase-CLGIPL, enzyme loading: 80.7 mg_enzme_/g_carrier_, 30°C, 1500 rpm, and pH 8.0.

For the next set of experiments, we turned our attention to naproxen malonate as starting material. The decarboxylation product, naproxen, is of high interest for the pharmaceutical industry. The enantioselective biosynthesis could be an advantage to design the process in a more environmentally friendly way. Due to the good solubility of naproxen malonate disodium salt (up to 200 mM at pH 8.0), we applied the substrate at 110 mM (30 g/L) in order to achieve high productivity in the intended process. We further investigated the enzyme’s specific activity without addition of buffer salts and observed only a slight shift in the pH and no significant loss of activity. Accordingly, a continuous back-titration for pH control was not necessary, which allows considerable savings in reagents and equipment. While using the immobilized purified AMDase-CLGIPL (on amino C2 acrylate carrier) full conversion to (*S*)-naproxen was achieved in six repetitive batches (**Figure 5**). The half-life of the free AMDase-CLGIPL in respect to naproxen malonate conversion (Supplementary Figure S5) could be determined as 1.2 h, which is the same value obtained from the aforementioned experiments with the wild type AMDase in phenylmalonate conversion (**Figure 2**). Thus, it states that the mutations done in the active site of the AMDase and the type of substrate used do not influence the enzyme’s process stability. Overall, in a real batch reaction time of up to 90 h, residual activity of the immobilized AMDase-CLGIPL enzyme could still be detected (**Figure 5**). Furthermore, this experiment demonstrates that the slightly reducing initial activities for the repetitive batches do not have a significant effect on the overall – macroscopic – outcome of the reaction under process conditions: full conversion can be easily reached even at prolonged exposition to process conditions.

### Optimization of the Immobilization Procedure by Using Cell Lysate

**FIGURE 6:**
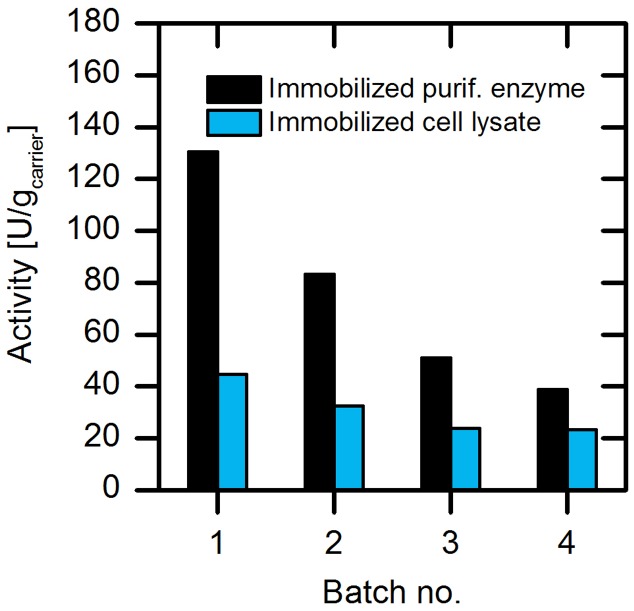
**Comparison of the activities of the (*S*)-selective AMDase-CLGIPL immobilized as cell lysate or as purified enzyme.** Reaction conditions: 50 mL reaction volume, 500 rpm, 30°C, 25 mM phenylmalonate, 4 g/L amino C2 acrylate carrier with immobilized purified form or cell lysate form of the (*S*)-selective AMDase-CLGIPL.

The efficiency of enzyme preparation is one crucial parameter for the application of enzymes in a production process. In this respect, enzyme purification is a major cost and time consuming factor, leading to fivefold higher production expenses compared to the application of the enzyme as crude cellular extract (Tufvesson et al., 2011). Hence, we further focused on the use of cell lysate instead of purified enzyme for immobilization strategies. In order to evaluate the usability of the cell lysate, we immobilized both the purified enzyme and the cell lysate on amino C2 acrylate carrier and tested their activity (**Figure 6**).

Our results revealed that per gram of preparation, the immobilized cell lysate exhibits 30% catalytic activity compared to the immobilized purified enzyme. When analyzing the amount of AMDase that is produced during protein expression, it was found to be about 30% of the total protein amount (determined based on the activity assay). Thus, the enzymatic activity from cell lysate coincides with the present AMDase content in respect to purified enzyme. In addition to that, we observed a reduced activity loss for immobilized cell lysate in repeated batch experiments. During four repetitive batches, the specific activity of the immobilized purified enzyme dropped by ∼70% from 130 to 40 U/g_carrier_, whereas the specific activity of immobilized cell lysate reduced 40% from 42 to 25 U/g_carrier_. AMDase seems to be more stable in a co-immobilized form with surrounding proteins from the cell lysate. This might be explained by a stabilizing effect caused by altered enzyme surface properties in presence of endogenous proteins from the cell lysate. This stabilizing effect of foreign proteins in the immobilization process is in some cases used to produce more stable enzyme preparations, for example by co-immobilization of helper proteins, like bovine serum albumin (BSA), in the production of cross-linked enzyme aggregates (CLEAs) (Ahumada et al., 2015).

### Investigation of the Process Stability of the Immobilized Cell Lysate

**FIGURE 7:**
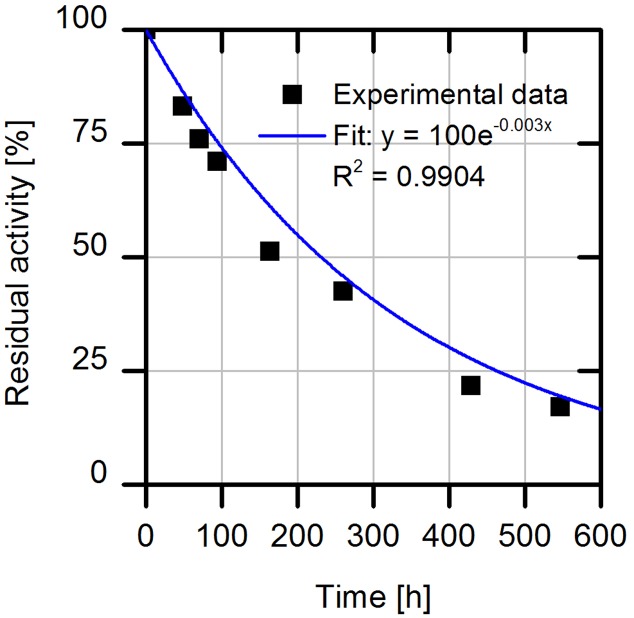
**Investigation of process stability of immobilized (*S*)-selective AMDase-CLGIPL on amino C2 acrylate carrier.** Reaction conditions: 1 mL reaction volume, 100 g/L amino C2 acrylate carrier with immobilized AMDase mutant, enzyme loading: 23.13 mg_enzyme_/g_carrier_, pH 8, 900 rpm, 30°C. Activity assay: conversion of 25 mM naproxen malonate. The carrier was washed after reaction with distilled water and incubated further in 1 mL water at pH 8.0, 30°C and 900 rpm.

In the last set of experiments, we evaluated the long-term operational stability of immobilized cell lysate on the amino C2 acrylate carrier. For this purpose, we incubated the enzyme formulation under process conditions at 30°C while shaking and measured residual enzyme activities by HPLC. We observed a half-life time of about 8.6 days (**Figure 7**), which corresponds to a 158-fold higher stability of immobilized AMDase-containing cell lysate compared to the free enzyme with a half-life time of only 1.2 h (determined for the wild type enzyme for phenylmalonate as substrate).

## Conclusion

In this study we successfully demonstrated the stabilization of AMDase by evaluating different solid supports with diverse characteristics. Next to the enzyme immobilization, we focused our attention on the optimization of the enzymatic process by eliminating the use of buffer salts as well as the purification step, which saves reagents and equipment. By these means, the (*S*)-selective AMDase-CLGIPL mutant is now qualified for a highly productive synthesis of arylpropionates. Our follow-up experiments will focus on the scaling-up of naproxen synthesis using the highly enantioselective (*ee* > 99%) (*S*)-AMDase variant.

## Author Contributions

MA: immobilization studies, preparation of the manuscript, approved the final version to be published. RK, LH, AL, SK: supervisor of the Ph.D. and Post-Docs, critical reading of the manuscript, approved the final version to be published. CM, SG, JE: designing the enzyme variant, activity studies, preparation and revision of the manuscript, approved the final version to be published.

## Conflict of Interest Statement

The authors declare that the research was conducted in the absence of any commercial or financial relationships that could be construed as a potential conflict of interest.
