# Supplementary material for: Improvement of the Process Stability of Arylmalonate Decarboxylase by Immobilization for Biocatalytic Profen Synthesis
Source: Front Microbiol. 2017 Mar 16;8:448. doi: 10.3389/fmicb.2017.00448 (PMC5352704; doi:10.3389/fmicb.2017.00448)
Supplement: Supplementary file 1 [file Data_Sheet_1.docx]

Supplementary Material

Improvement of the process stability of arylmalonate decarboxylase by immobilization for biocatalytic profen synthesis

M. Aßmann^1^, C. Mügge^2^, S. K. Gaßmeyer^2^, J. Enoki^2^, L. Hilterhaus^1^, R. Kourist^2^, **A. Liese^1*^ and S. Kara^1*^**

^1^Institute of Technical Biocatalysis, Hamburg University of Technology, Denickestrasse 15, 21073 Hamburg, Germany.

^2^Junior Research Group for Microbial Biotechnology, Ruhr-University Bochum, 44780 Bochum, Germany.

*** Correspondence:**Dr. Selin Kara, Institute of Technical Biocatalysis, Hamburg University of Technology, Denickestrasse 15, 21073 Hamburg, Germany, [selin.kara@tuhh.de](mailto:selin.kara@tuhh.de)

Prof. Dr. Andreas Liese, Institute of Technical Biocatalysis, Hamburg University of Technology, Denickestrasse 15, 21073 Hamburg, Germany, [liese@tuhh.de](mailto:liese@tuhh.de)

Table S1: Carrier properties according to suppliers’ specifications.

| **Carrier** | **Linkage** | **Functionality (density [µmol/g])** | **Matrix**  **(additional coating)** | **Particle diameter [µm]** | **Pore diameter [nm]** |
| --- | --- | --- | --- | --- | --- |
| Amino C2 acrylate | covalent | Amino (1760) | acrylate | 423 | 119.5 |
| Sepabeads EC-EP | covalent | Epoxy (>100) | polymethacrylate | 200–500 | 10–20 |
| Sepabeads EC-HA | covalent | Hexamethyl-amino (>700) | polymethacrylate | 200–500 | 10–20 |
| Trisoperl® Porous glass beads | adsorption | / | porous glass | 100–200 | 120 |
| Trisoperl® amino | adsorption | / | porous glass | 100­–200 | 120 |
| EziG1^TM^ | complex | Co(II) or Fe(III) (>10) | porous glass  (longchain aminoalkyl) | 74–125 | 50 |
| EziG2 ^TM^ | complex | Co(II) or Fe(III)  (>10) | porous glass (vinylbenzylchloride) | 74–125 | 50 |
| EziG3 ^TM^ | complex | Co(II) or Fe(III)  (>10) | porous glass  (copolymer styrol and acrylnitrile) | 74–125 | 50 |

**Calculation of the initial activity in repeated batch experiments**

For initial activity measurement, the data of the product formation was fitted in Origin 8.5.1 with the Michaelis-Menten equation. The fitted data were used to calculate the conversion. A value with ~5‑6 % conversion was selected to ensure a determination within the initial phase of the reaction.


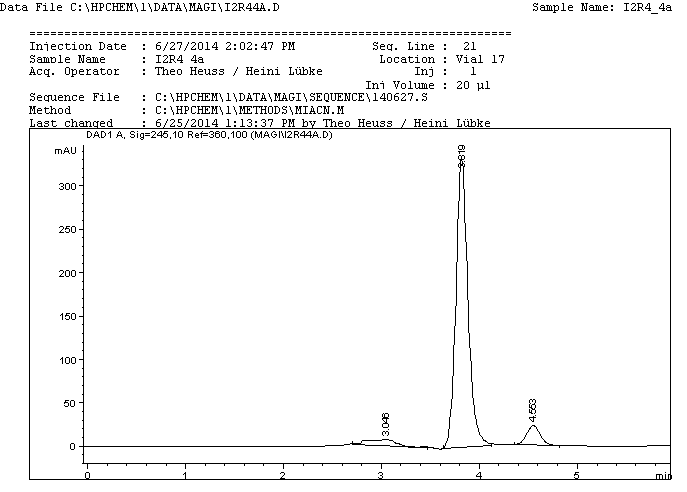


Phenylmalonate

Phenylacetic acid

Figure S1: A reference HPLC chromatogram of phenylmalonate and phenylacetic acid. HPLC analysis was realized with a C18 reversed phase column (Nucleodur C18 pyramid 250/4.6, Machery Nagel) in an Agilent 1100 HPLC system using an isocratic eluent of ACN:H_2_O:TFA (59.025:39.025:0.05). The flow rate of 0.8 mL/min was maintained for 6 min. The detection was carried out with a diode array detector at a wavelength of 245 nm.


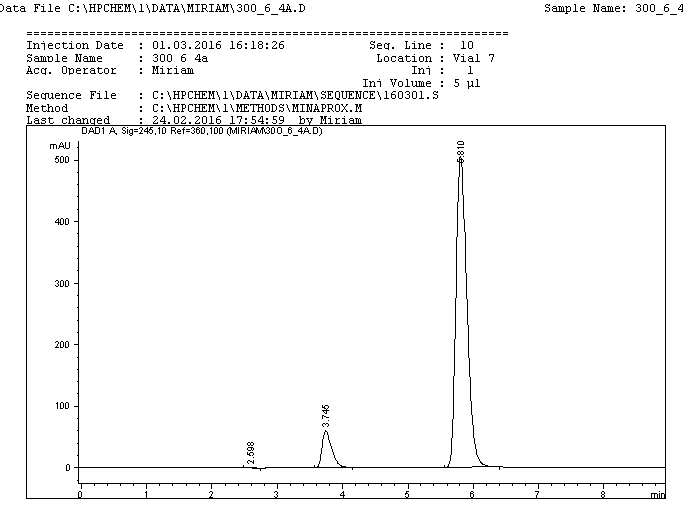


Naproxen

Naproxen malonate

Figure S2: A reference HPLC chromatogram of naproxen malonate and naproxen. HPLC analysis was realized with a C18 reversed phase column (Nucleodur C18 pyramid 250/4.6, Machery Nagel) in an Agilent 1100 HPLC system using an isocratic eluent of ACN:H_2_O:TFA (59.025:39.025:0.05). The flow rate of 0.8 mL/min was maintained for 9 min. The detection was carried out with a diode array detector at a wavelength of 245 nm.

Structure of wildtype AMDase


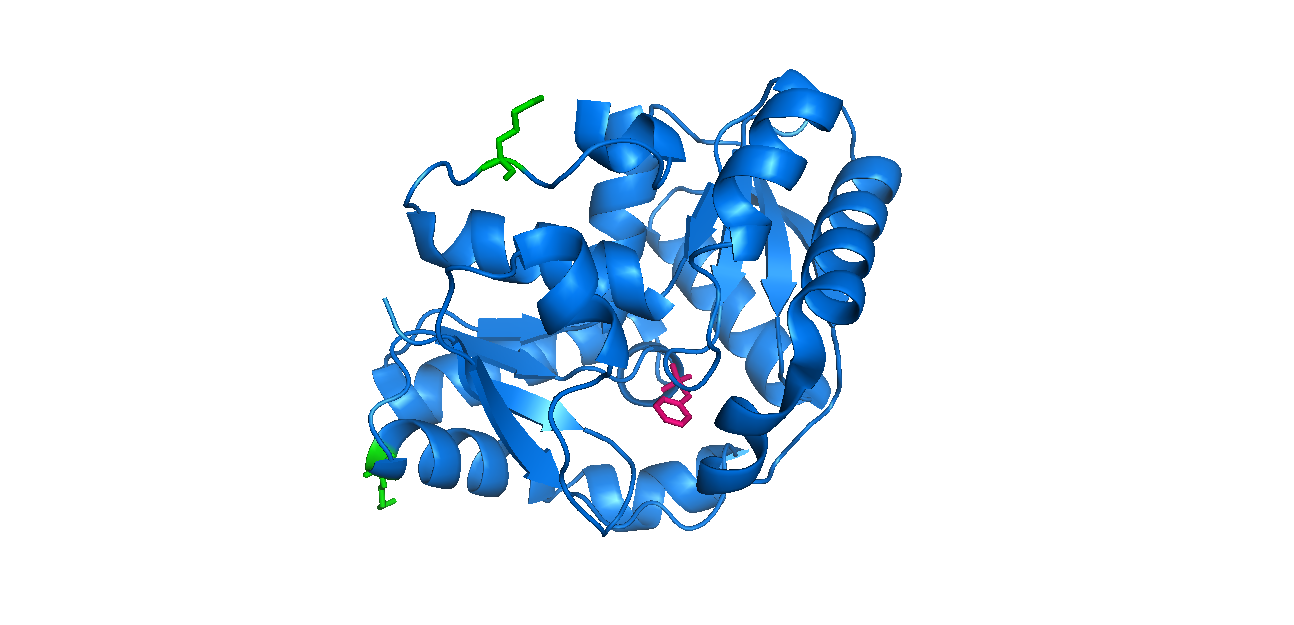

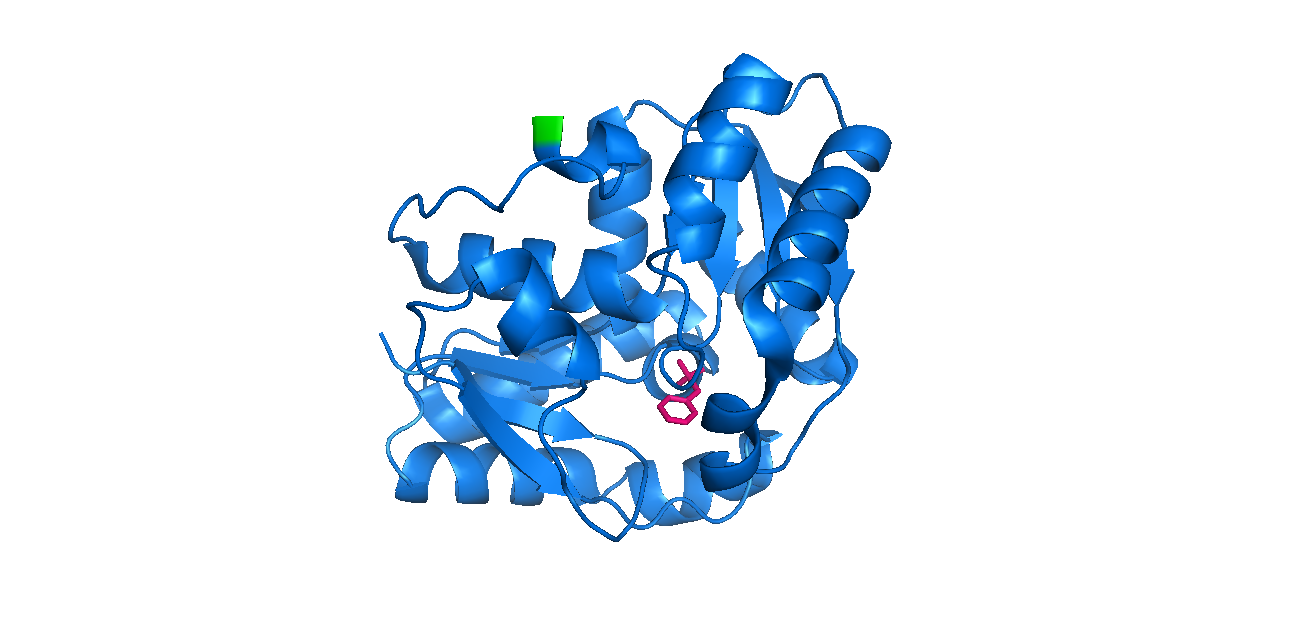


Figure S3: Structure of the wildtype AMDase based on the free available PDB file 3IP8 in complex with benzylphosphonate in the active side (marked in magenta) (Okrasa et al. 2009). Left: Lysine residues are marked in green; right: the N-terminus (position of the His_6_ Tag) of the enzyme is marked in green.

**Kinetic characterization of purified free AMDase (wild type and the mutant)**

| **(A)** | **(B)** |
| --- | --- |
| **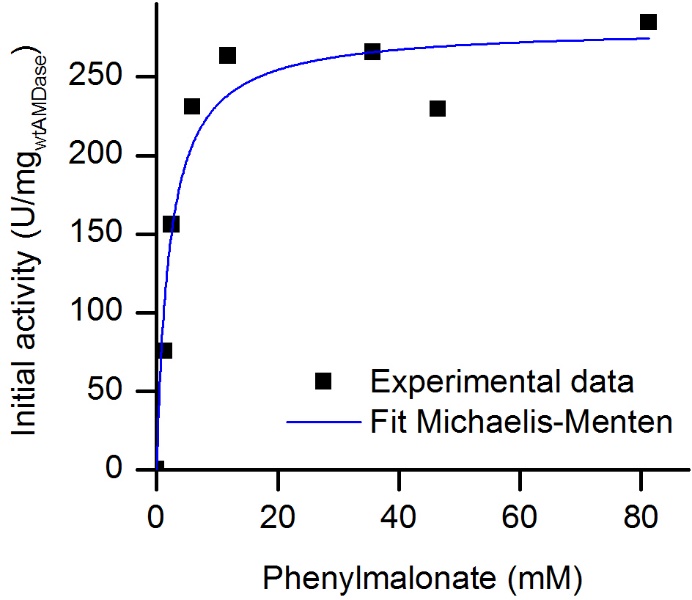** | **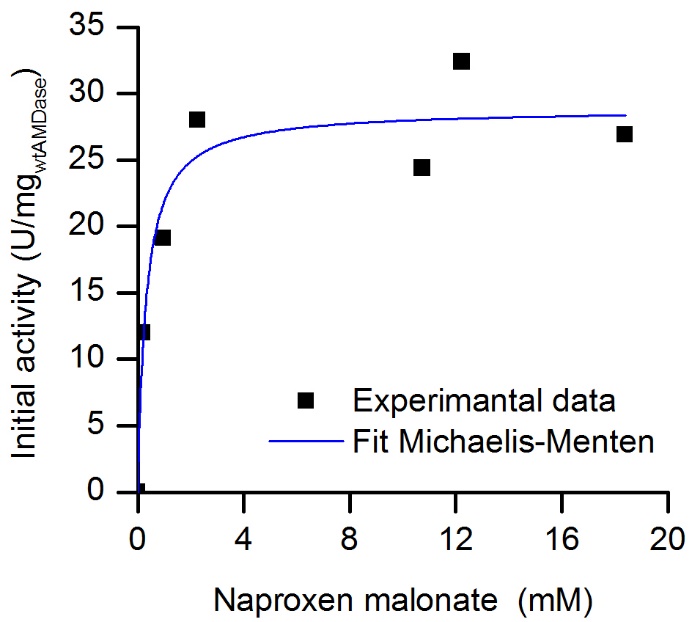** |
| **(C)** | **(D)** |
| **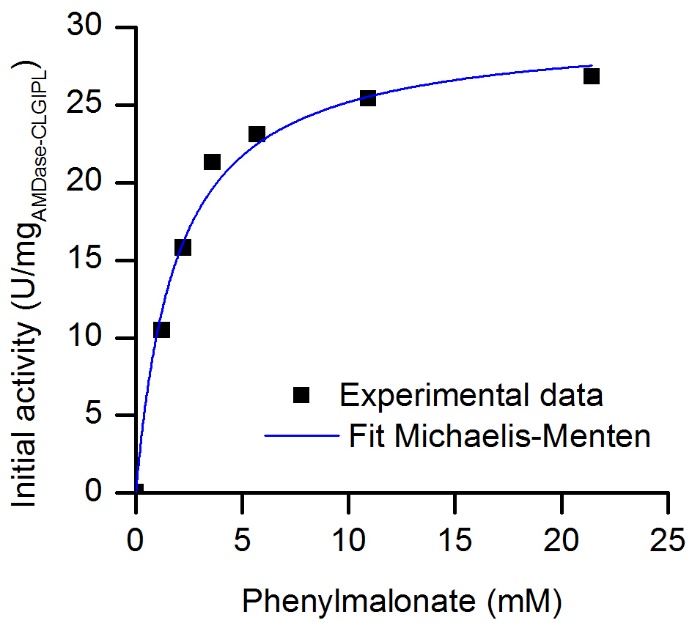** | **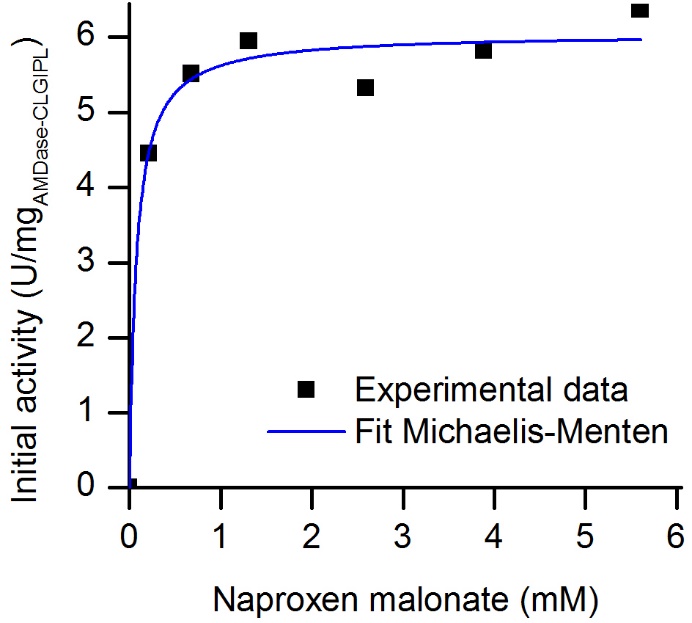** |

**Figure S4:** Kinetic analysis of the free purified wtAMDase and AMDase-CLGIPL in the conversion of the model substrates phenylmalonate (PM) and naproxen malonate (NM). Reaction conditions: 500 µL volume, 30°C, 500 rpm and (A): [PM] = 0–80 mM (0.08 µM wtAMDase); (B): [NM] = 0–18 mM (0.4 µM wtAMDase), (C): [PM] = 0–21 mM (0.4 µM AMDase-CLGIPL) (D) [NM] = 0–6 mM (0.4 µM AMDase-CLGIPL). The kinetic parameters were calculated by fitting the data to the one-substrate Michaelis-Menten kinetic with Origin 8.5.1 and are given in Table S2.

Table S2: Kinetic parameters of the wtAMDase and AMDase-CLGIPL in respect to phenylmalonate and naproxen malonate conversion.

| **Enzyme** | **Substrate** | **V_max_ [U/mg]** | **K_M_ [mM]** |
| --- | --- | --- | --- |
| wtAMDase | Phenylmalonate | 281.7 ± 17.5 | 2.15 ± 0.67 |
| wtAMDase | Naproxen malonate | 28.85 ± 2.06 | 0.32 ± 0.15 |
| AMDase-CLGIPL | Phenylmalonate | 29.98 ± 1.5 | 1.91 ± 0.29 |
| AMDase-CLGIPL | Naproxen malonate | 6.04 ± 0.21 | 0.075 ± 0.027 |

**Residual activity of the free AMDase-CLGIPL in the naproxen malonate conversion**

| 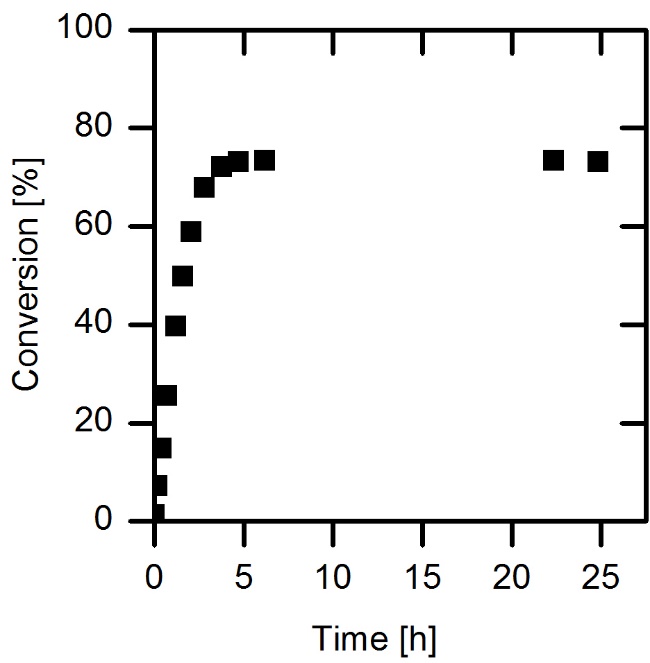 | 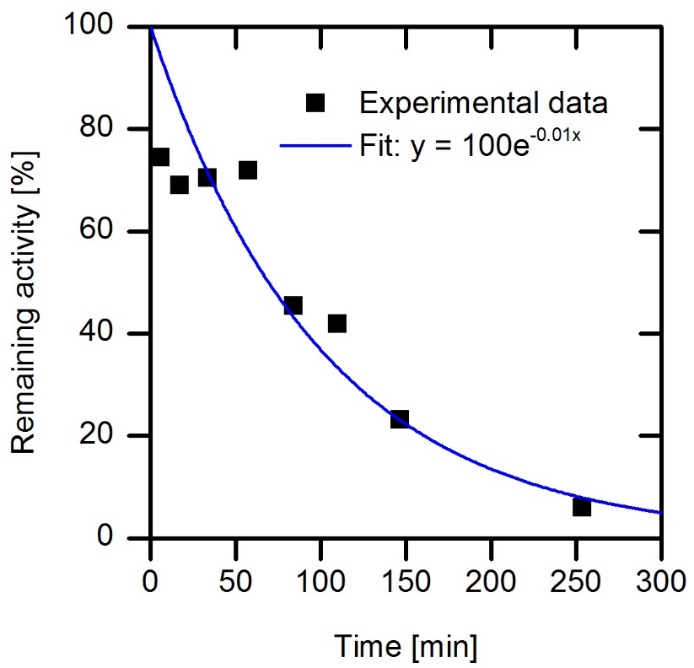 |
| --- | --- |

Figure S5: Conversion of naproxen malonate catalyzed by purified free enzyme (left) and residual activity (right). Reaction conditions: 1 mL reaction volume, 120 mM naproxen malonate, 100 mg/L purified free (*S*)-selective AMDase-CLGIPL, 30°C, 750 rpm, pH 8.0.

**Determination of the AMDase content in cell lysate**

The amount of the target AMDase enzyme found in the cell free extract was determined based on an activity assay by comparing the activities of the purified enzyme and with the cell free extract under the same conditions. Assay conditions: 20 mM phenymalonate, 10% (v/v) purified enzyme or cell lysate (both previously 100-fold diluted and the protein concentration measured *via* Bradford assay) in ultra-pure water at pH 8.0, 30°C, and 500 rpm in 0.5 mL scale. The activity results show that ~30% of the cell free extract contains the target enzyme.

An example of the SDS-PAGE analysis is shown below (Figure S6).


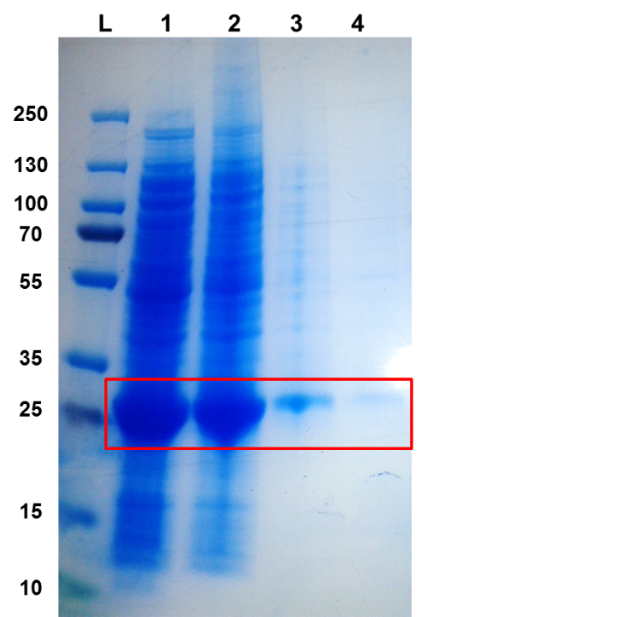


Figure S6: SDS-PAGE of the cell lysate before and after immobilization. L (Ladder): Thermo Scientific PageRuler Plus Prestained Protein Ladder; 1: cell lysate (39 µg_protein_/lane); 2: cell lysate recovered after immobilization (14 µg_protein_/lane); 3: washing step with water (3 µg_protein_/lane); 4: washing step with 0.5 M NaCl (0.3 µg_protein_/lane). AMDase: 25 kDa.

**Analysis of the phenylmalonate adsorption on EziG-1 Co(II) and amino C2 acrylate carrier**

**Figure S7:** Analysis of phenylmalonate adsorption on EziG-1 Co(II) and amino C2 acrylate carriers. Conditions: 1.5 mL reaction volume, 50 mg carrier, 20 mM phenylmalonate, pH 7, 40 mM Tris, 30°C, 200 rpm, incubation for 19 h. Phenylmalonate: content before incubation (corresponds to 100%).

References

Okrasa, K., Levy, C., Wilding, M., Goodall, M., Baudendistel, N., Hauer, B., Leys, D. & Micklefield, J. 2009. Structure-guided directed evolution of alkenyl and arylmalonate decarboxylases. *Angewandte Chemie (International ed. in English)* **48**, 7691–7694.
